# Supplementary material for: Unveiling the Role of the Work Environment in the Quality of Life of Menopausal Physicians and Nurses
Source: Int J Environ Res Public Health. 2023 Sep 12;20(18):6744. doi: 10.3390/ijerph20186744 (PMC10531365; doi:10.3390/ijerph20186744)
Supplement: Supplementary file 1 [file ijerph-20-06744-s001.zip › ijerph-2522832-supplementary.pdf]

Supplementary Materials

Table S1. Proportion of participants with menopausal symptoms at different age groups

Symptom	Age categories			p-value
	40-49 yrs.	50-54 yrs.	55+ yrs.	
**Vasomotor symptoms**				
Hot flashes	32.76%	56.1%	41.38%	0.068
Nights sweats	26.32%	47.62%	34.48%	0.09
Sweating	27.59%	57.14%	27.59%	0.005
**Psychological symptoms**				
Being dissatisfied with their personal life	32.14%	43.9%	33.33%	0.459
Feeling anxious or nervous	49.09%	56.1%	34.48%	0.199
Experiencing poor memory	51.79%	58.54%	39.29%	0.289
Accomplishing less than they used to	45.45%	41.03%	49.67%	0.875
Feeling depressed, down or blue	45.45%	50%	23.33%	0.062
Being impatient with other people	51.85%	56.41%	31.03%	0.092
Feelings of wanting to be alone	44.44%	45.24%	35.71%	0.692
**Physical symptoms**				
Flatulence or gas pains	57.14%	57.14%	46.67%	0.601
Aches in the muscles and joints	56.6%	58.54%	53.57%	0.92
Feeling tired or worn out	71.7%	74.36%	77.78%	0.84
Difficulty sleeping	43.64%	63.41%	60.71%	0.114
Aches in the back, neck or head	61.11%	64.29%	66.67%	0.878
Decrease in physical strength	60%	72.5%	64.29%	0.449
Decrease in stamina	63.64%	65%	72.41%	0.709
Feeling a lack of energy	66.04%	68.29%	65.52%	0.963
Dry skin	66.04%	64.29%	68.97%	0.919
Weight gain	47.27%	65.85%	66.67%	0.102
Increased facial hair	17.86%	26.83%	37.93%	0.127
Changes in appearance, texture or tone of skin	55.56%	53.66%	58.62%	0.919
Feeling bloated	49.09%	53.66%	46.67%	0.831
Low backache	68.63%	54.76%	51.72%	0.236
Frequent urination	40%	45.24%	28.57%	0.371
Involuntary urination when laughing or coughing	27.78%	31.71%	44.83%	0.282
**Sexual symptoms**				
Change in sexual desire	33.93%	50%	39.29%	0.273
Vaginal dryness during intimacy	24.56%	33.33%	50%	0.064
Avoiding intimacy	41.82%	52.5%	55.56%	0.411
